# Morphic Sensors for Respiratory Parameters Estimation: Validation against Overnight Polysomnography

**DOI:** 10.3390/bios13070703

**Published:** 2023-07-03

**Authors:** Ganesh R. Naik, Paul P. Breen, Titus Jayarathna, Benjamin K. Tong, Danny J. Eckert, Gaetano D. Gargiulo

**Affiliations:** 1 Adelaide Institute for Sleep Health (Flinders Health and Medical Research Institute: Sleep Health), College of Medicine and Public Health, Flinders University, Bedford Park, SA 5042, Australia; danny.eckert@flinders.edu.au; 2 College of Science and Engineering, Flinders University, Bedford Park, SA 5042, Australia; 3 The MARCS Institute, Western Sydney University, Westmead, NSW 2145, Australia; p.breen@westernsydney.edu.au (P.P.B.); titus.jayarathna@westernsydney.edu.au (T.J.); g.gargiulo@westernsydney.edu.au (G.D.G.); 4 Neuroscience Research Australia, Randwick, NSW 2031, Australia; b.tong@neura.edu.au; 5 Sleep Research Group, Charles Perkins Centre, School of Medicine, University of Sydney, Camperdown, NSW 2006, Australia; 6 School of Engineering, Design and Built Environment, Western Sydney University, Penrith, NSW 2751, Australia

**Keywords:** morphic sensor, respiratory rate, heart rate, wearables, polysomnography

## Abstract

Effective monitoring of respiratory disturbances during sleep requires a sensor capable of accurately capturing chest movements or airflow displacement. Gold-standard monitoring of sleep and breathing through polysomnography achieves this task through dedicated chest/abdomen bands, thermistors, and nasal flow sensors, and more detailed physiology, evaluations via a nasal mask, pneumotachograph, and airway pressure sensors. However, these measurement approaches can be invasive and time-consuming to perform and analyze. This work compares the performance of a non-invasive wearable stretchable morphic sensor, which does not require direct skin contact, embedded in a t-shirt worn by 32 volunteer participants (26 males, 6 females) with sleep-disordered breathing who performed a detailed, overnight in-laboratory sleep study. Direct comparison of computed respiratory parameters from morphic sensors versus traditional polysomnography had approximately 95% (95 ± 0.7) accuracy. These findings confirm that novel wearable morphic sensors provide a viable alternative to non-invasively and simultaneously capture respiratory rate and chest and abdominal motions.

## 1. Introduction

Wearable devices and flexible sensors have been widely used and investigated as an emerging technology platform to continuously monitor vital human signs during daily life and in various clinical applications [1,2,3,4]. Several studies have demonstrated that regular monitoring of critical body parameters such as blood pressure, heart rate (HR), body temperature, respiratory rate (RR), and inter-breath interval (IBI) variability can be highly informative for a range of clinical diagnoses [5,6,7,8]. While multiple technological solutions can provide continuous HR monitoring, only a few can provide continuous RR and IBI monitoring. Continuous RR and IBI monitoring may improve asthma, pneumonia, chronic obstructive pulmonary disease (COPD), hypertension, and sleep apnea diagnosis and monitoring [8,9,10,11]. Respiration can be detected via rhythmic chest/abdomen movement or the air moving in/out of the nostrils or mouth. Monitoring changes in respiratory parameters can be used to detect sleep-disordered breathing events such as snoring, obstructive sleep apnea, central sleep apnea (CSA), and hypoventilation syndromes [12,13,14]. However, the utility of respiratory parameters (RR and IBI) extends beyond sleep. For example, elevated RR is a primary predictor of clinical deterioration within two days of discharge from the emergency department [15]. Similarly, IBI (breathing variability) at rest is positively associated with 24 h blood pressure [11].

Modern RR monitoring technology includes pulse oximetry, which uses infrared light and requires a probe linked to the patient’s finger [16]. Some researchers have used the electrocardiography (ECG)-derived method to estimate RR, which typically also requires an electrode connection on the patient’s body [17]. Radiofrequency (RF)-based RR monitoring approaches have been proposed in several studies. For instance, Abdelnasser et al. [18] proposed UbiBreathe, a ubiquitous non-invasive WiFi-based breathing estimator for sleep research. Adib et al. [19] integrated breathing and heart rate estimators using smart home monitors. Liu et al. [20] proposed a method for tracking vital sleep signs using off-the-shelf WiFi. In another study, Ravichandran et al. [21] presented Wibreathe, a wireless device designed to estimate respiration in the home environment. In these approaches, RR is estimated by capturing the variation in the wireless signal’s channel information caused by chest movement during respiration.

Although in-laboratory polysomnography remains the gold standard for objective sleep monitoring, at-home sensor systems have recently gained popularity. In polysomnography, respiration is measured by nasal airflow and chest wall movements. This includes sensors worn on the face or abdomen, which can potentially disrupt sleep [22]. In-laboratory polysomnography requires specially designed facilities, high-cost proprietary wearable sensors, and professional installation by trained staff. This level of resourcing is impractical for long-term sleep monitoring and is limited to specialty clinical and research use [23]. Watanabe and Watanabe [24] attempted to solve this problem by using a mattress that measures heart rate and breath count. The system is unobtrusive. However, it requires sensor installation on the bed, and the detection accuracies are affected by the user’s height and weight [25].

In-laboratory polysomnography may disrupt the same sleep parameters one intends to measure. Furthermore, at-home sensing allows tracking vital sleep signs such as respiratory and cardiac parameters over time, which is critical to detect night-to-night variability, whether stochastic or linked to waking behaviors or exposures that vary over time [26]. Our study focuses on the assessment of respiratory parameters in people with sleep apnea using in-house built and inexpensive morphic sensors. Our study incorporated wearable, non-invasive, noncontact, and unobtrusive sensors (embedded in a standard t-shirt) based on three electro-resistive bands [27]. This paper explains the design and instrumentation of the sensor, the implementation, evaluation, and comparison of cardiorespiratory parameters (RR, HR) with gold standard polysomnography airflow measurement.

## 2. Morphic Sensors

During the past few years, the morphic sensor has evolved since its first inception in 2015 [28]. This section briefly describes the third-generation electro-resistive band (ERB) morphic sensor, which includes a complete redesign of the electronics and garment.

**Figure 1:**
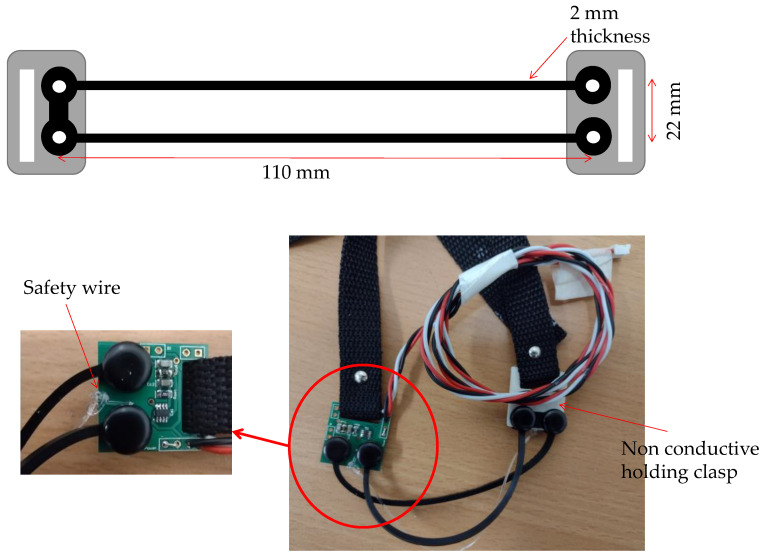
Electro-resistive band (ERB) assembly. (**Top**) ERB. (**Left**) Front-end PCB. (**Right**) Wiring loom, clasps, and straps.

ERBs require polarisation and signal conditioning. Like our previous designs [27,28], each ERB is polarized using a small DC current achieved using a dedicated current generator LT3092 by Linear Technology (Linear Technologies, New York, NY, USA). Due to the setting constraints of the LT3092, the polarization current is fixed at 500 µA. The elicited voltage changes due to the ERB stretching are amplified with a gain of 2 *v*/*v* by a first-order active lowpass filter designed for a precision operational amplifier (OPA140 (Texas Instruments, Dallas, TX, USA, 2015)). The cut-off frequency is set to 500 Hz to comply with the sampling rate of 1 kHz used. Although the ERB sensors are not in direct contact with the skin; this sensor does not require skin contact. As the polarization current value is higher than the micro-shock hazard (J. G. Webster, 2009), to ensure user safety, the final ERB assembly was enclosed into an isolating sleeve that was subsequently sewn on the outside of a loose-fit t-shirt. The Bespoke ERB is carbon-doped silicone rubber (Shore hardness 45A) cast into a “U-shaped” form factor with clasp/circuit connection eyelets manufactured to have a base impedance of 1 kΩ (Figure 1). The four eyelets connect the ERB directly to the signal conditioning circuit fabricated on a small dual-layer printed circuit board. Being U-shaped, there is no need for a return wire. To avoid the overstretching and ripping of the ERB, a thin safety line matching the maximum permitted stretching for the ERB is affixed to the printed circuit board and a holding clasp on the other side (Figure 1). The maximum permitted elongation for the ERB sensor was determined using a bespoke stress testing device [22,29,30]. The stress testing device enabled the determination of the sensor breaking point and was also employed to characterise the sensor and fine-tune the circuit parameters leading to this new front end. A direct comparison between the bills of material for the current and previously used front ends is reported in Table 1.

**Figure 2:**
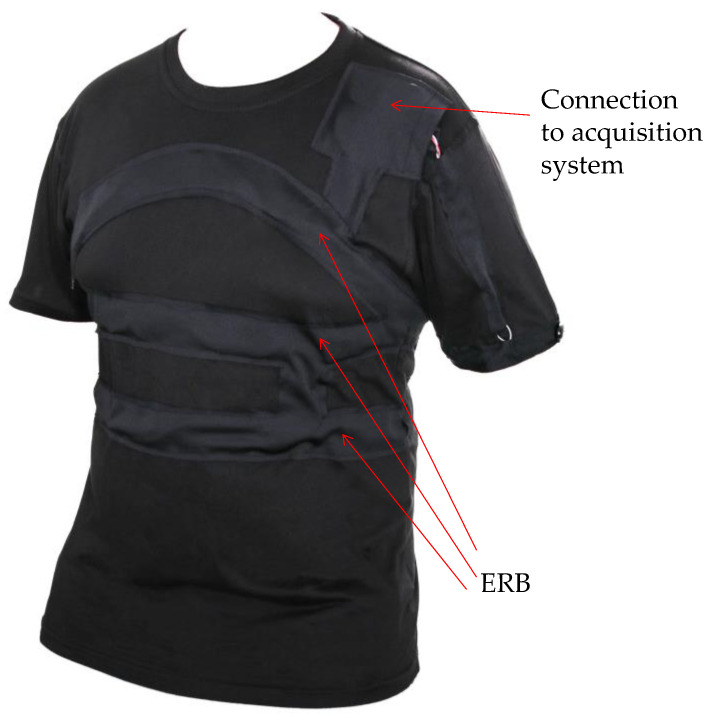
Third-generation ERB morphic sensors fully assembled in a t-shirt (image courtesy of Medical Monitoring Solutions Pty Ltd., Sydney, Australia).

Like the previous versions, the morphic sensor exploits the electro-resistive nature of the material, whereby resistance varies with the stretching impressed to the sensor by the moving ribcage during respiration. Resistance variations are transformed into voltage variations by the polarization circuit. To cover the entire chest area, for this application, we employed three ERBs (see Figure 2). Summation of ERB voltages (un-calibrated chest volume signal) is achieved during post processing. The three wire connections from each ERB front end are threaded into a dedicated pocket positioned not to obstruct the other sensors necessary for sleep monitoring. Connection with the acquisition system and the sensor power supply is achieved via a multicore flexible shielded cable threaded through the dedicated wall data interface shared with the polysomnography system. The t-shirt is designed to be worn over the top of the user’s clothing, sensor fitting, with specific wearer adjustment, and ERB tension is achieved via securing the non-conductive ERB harness which, once worn, should be snug but not restrictive.

## 3. Methods

### 3.1. Participants

Thirty-two people (26 male and 6 female) with previously diagnosed sleep apnoea (via overnight polysomnography) volunteered to wear the morphic sensor during a research polysomnography study. Inclusion criteria were: (i) 18–70 yr and (ii) apnea-hypopnea index (AHI) > 10 events/h sleep. Exclusion criteria were: (i) cardiovascular disease, (ii) neurological disorder, and (iii) pregnant or breastfeeding women. The anthropometric data of the study participants are summarized in Table 2. Ethical approval was obtained from the Southeastern Sydney Local Health District (HREC No. 16/356) and the Western Sydney University Human Research Ethics Committees. All participants provided written informed consent.

### 3.2. Data Collection

Sleep data were collected from patients using both polysomnography (Philips Respironics Alice 6 LDx 31 channel sleep data acquisition system) and the ERB embedded t-shirt (Figure 2). The t-shirt design allows the topmost band to be positioned below the axilla while the bottom band is just below the diaphragm. This configuration allows for full coverage of the thoracic cage. The t-shirt was worn for the entire overnight sleep study, and the respiratory-related information was recorded with a sampling rate of 1 kHz. Polysomnography-derived nasal airflow was also recorded at a 1 kHz sampling rate.

## 4. Data Analysis

### 4.1. Pre-Processing, Artifact Removal (Groundtruth GUI)

Data from the neuromorphic sensors and airflow polysomnography data are contaminated by artifacts generated due to participants’ body movements. Data were filtered using a lowpass filter with a cut-off frequency of 1 Hz. Respiration peaks were initially detected using the inbuilt window-based threshold method (Peak Detect). However, automated peak detection identifies more features in the data than those of interest (i.e., respiration cycles). Hence, incorrect features (peaks) were removed using our own “Groundtruth GUI” MATLAB software developed for artifact and feature identification of physiological data [31]. Pre-processed (Groundtruth GUI) clean morphic sensors and polysomnography sensor respiration data were considered for respiratory parameters calculations.

### 4.2. Respiratory Rate

Instantaneous breath-by-breath respiratory rates were obtained from the position of two consecutive respiratory peaks (*P_i_* and *P_j_*) and the sampling rate (*F_S_* = 1000 Hz). The time difference Td between the two peaks is calculated using the following formula:

(1) $$T_{d}=\frac{\left|P_{i}-P_{j}\right|}{F_{s}}$$

**Figure 3:**
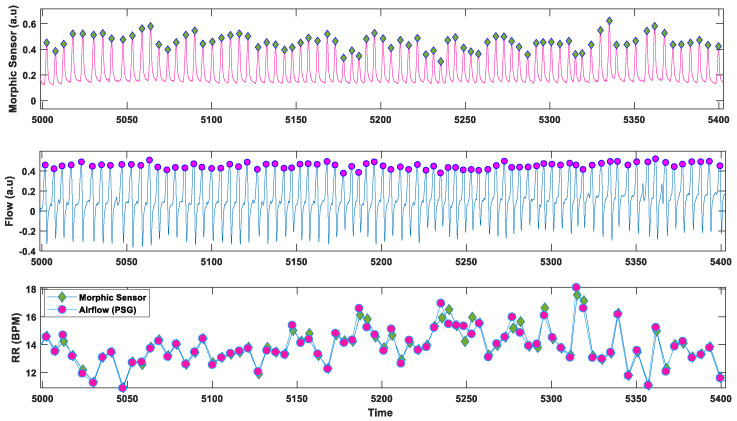
An example of respiratory rate (RR) plot of morphic sensor and airflow (polysomnography): third axis showing RR instantaneous RR for morphic sensor and airflow (polysomnography).

The instantaneous respiration rate (FR) from both raw and pre-processed (using Groundtruth GUI) data were then calculated by taking the inverse of T_(d,), i.e., FR = 1/*T_d_*. The respiration rate was then calculated for 60 s intervals (breaths/min) and averaged (multiple 60 s intervals) over the entire data length. An example of the RR derived from the morphic sensor and polysomnography airflow signal in one participant is shown in Figure 3.

### 4.3. Inter-breath Interval Variability

A healthy individual’s standard respiratory rhythm can be seen during normal breathing, with a relatively constant RR and tidal volume. However, any variations within this respiratory rhythm are characterised as inter-breath interval (IBI) variability [32]. Changes in RR are a vital indicator that often precedes major clinical manifestations of serious complications, such as respiratory depression and respiratory tract infections. Marked changes in IBI, (the period in seconds between 2 consecutive peaks—the interval between successive breaths), is a surrogate marker of adverse outcomes in multiple pathological states. For example, sepsis [33,34].

## 5. Results

The individual results for RR calculated after pre-processing (using Groundtruth GUI) are reported in Table 3. Similarly, the results for IBI variability after pre-processing (using Groundtruth GUI) are reported in Table 4. Data are summarized as mean ± SD.

### Statistical Analysis—Bland–Altman Evaluation

The Bland–Altman method was used to determine bias (mean difference between methods) and limits of agreement (±95% confidence around bias) to compare RR from original and Groundtruth GUI processed data. This method provides a quantitative and visual comparison of two measurements by plotting the differences between the two techniques against the average of the two methods. It supplements correlational analyses by determining the limits of agreement between methods (in which the mean level ±1.96 SD represents a bias ±95% confidence interval range of agreement).

**Figure 4:**
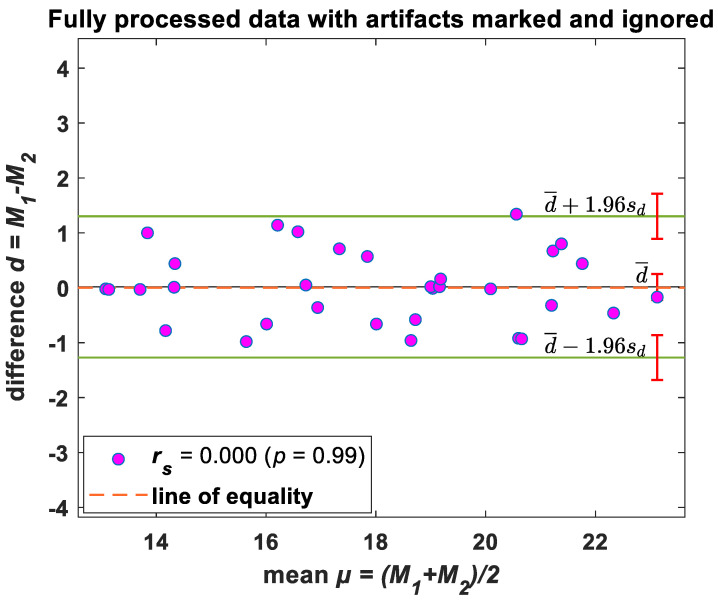
Bland–Altman plots—RR. Difference between windowed morphic sensor data and polysomnography airflow breaths/min plotted against the average value of the two methods. Solid horizontal lines indicate mean difference and upper/lower limits of agreement; sd: standard deviation. Note: Please refer to Figure S1 for the Bland–Altman plot of unprocessed (raw) data.

The Bland–Altman plot (Figure 4) showed that the mean difference between the morphic sensor and the polysomnography flow for the Groundtruth GUI processed (cleaned data) data was negligible with a narrow confidence interval (95% agreement limit −1.27, 1.30; Spearman rank correlation between mean and difference 0.003), indicating a high degree of agreement between the derived estimates from the two sensors.

**Figure 5:**
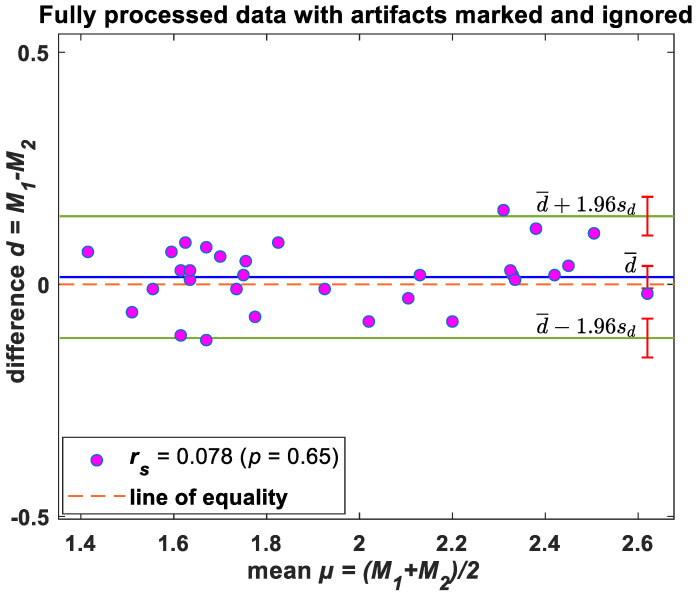
Bland–Altman plots—IBI variability. Difference between windowed morphic sensor data and polysomnography airflow breaths/min plotted against the average value of the two methods. Solid horizontal lines indicate mean difference and upper/lower limits of agreement; sd: standard deviation. Note: Please refer to Figure S2 for the Bland–Altman plot of unprocessed (raw) data.

Similarly, Bland–Altman results for the IBI variability values displayed in Figure 5 showed that the mean difference between the morphic sensor and the polysomnography airflow for the Groundtruth GUI processed (cleaned data) data was small at 0.13 breaths/min (95% agreement limit—0.116, 0.147; Spearman rank correlation between mean and difference 0.0845).

**Figure 6:**
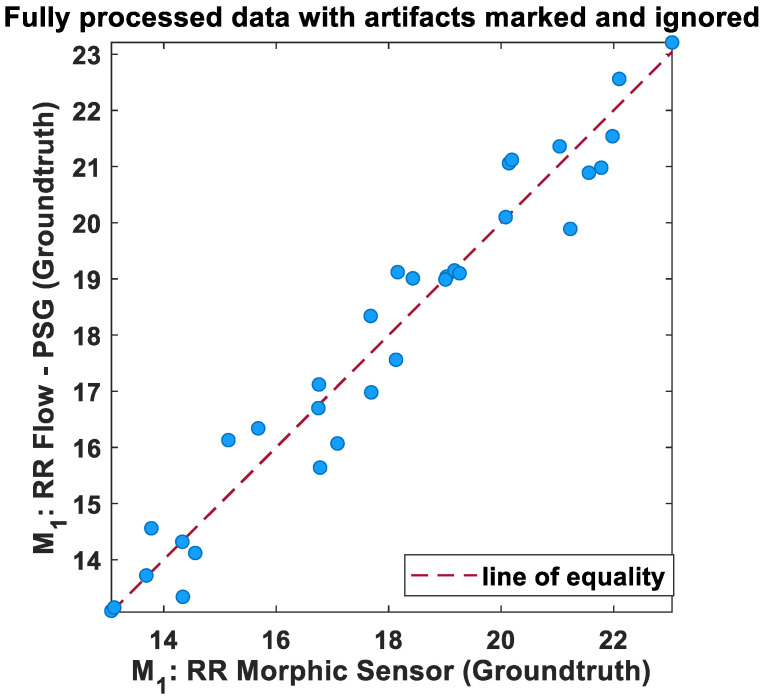
Regression plot of the mean RR of morphic sensors and polysomnography airflow (breaths/min). The dotted lines indicate the regression line.

The relationship between averaged values of RR computed from morphic sensors and polysomnography, computed over 60 s intervals, was also assessed using a linear regression scatter plot. The scatter plot of RR and the processed data is shown in Figure 6. Ideally, the two techniques would exhibit perfect agreement, and when the data is plotted as a scatter plot, all points will lie on the line of equality. From the results, the two methods are in good agreement following Groundtruth analysis.

**Figure 7:**
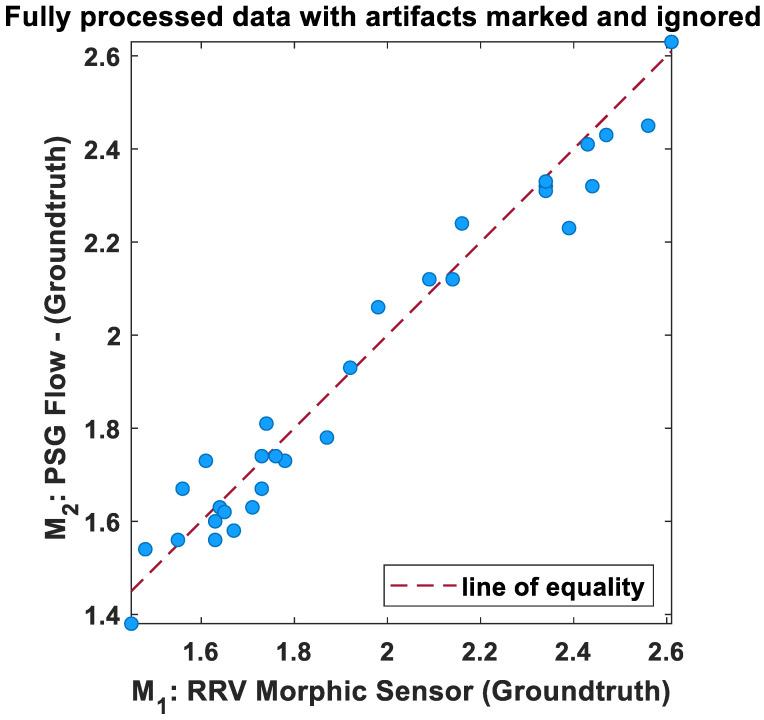
Regression plot of the mean IBI variability of morphic sensors and polysomnography airflow (breaths/min). The dotted lines indicate the regression line.

The scatter plot of IBI variability for processed data is shown in Figure 7. From the results, the two methods are in good agreement following pre-processing of data (Groundtruth analysis).

## 6. Discussion

The use of wearable devices to monitor breathing activity has made a pathway for many new medical services, enabling remote, accurate, and local monitoring of a patient’s conditions. Many wearable sensors have been proposed in the literature and are on the market to detect respiratory parameters using various methods, including chest movements, exhaled gases, blood oxygenation changes, and chest sounds. Likewise, both time and frequency domain signal processing methods have been reported as capable of extracting useful information from the various sensors.

The respiratory parameters (RR and IBI) computed from our proposed method represent a more efficient and potentially more tolerable approach for overnight polysomnography. In Table 5, we compare our results with similar methods reported in the literature regarding sensor types, signal processing techniques, respiratory parameter computation, and efficiency.

From Table 5, we can infer that the time domain methods, such as [35,36,42], depend on analyzing the acceleration trends for detecting the peaks related to the respiratory movements, offering good performance accuracy and low computational complexity. However, the detected acceleration waveforms are subject to artefacts due to body movements and other physiological signals with a size comparable to those associated with breathing, inducing potential errors in RR measurement [43]. The frequency domain approaches [37,41,44], based on popular FFT-based estimators and peak detection, represent an alternative solution, allowing for a straightforward reduction of motion-induced artefacts, but requiring a higher computational load [45]. Our proposed method uses the artefact detection method “Groundtruth”, followed by simple peak detection to compute RR parameters. The method is reliable and consistent for the duration of an entire overnight sleep study. Moreover, our approach reports IBI from RR, which is the first study of its kind and allows for the potential to extract cardiac parameters simultaneously accurately from the morphic sensors.

## 7. Conclusions

Wearables and sleep trackers are gathering traction among the public, often due to their seamless integration with personalized electronic devices. Wearables offer more feasible and reliable alternatives to measure sleep and cardiorespiratory patterns compared to traditional in-laboratory approaches. Wearable devices are revolutionizing how we treat, manage, and prevent diseases, enabling integrated and accurate monitoring of the patient’s health, with the potential to lower management costs and provide more accurate and timely diagnosis, including early prevention, continuous tracking, and quicker intervention. Monitoring respiratory activity is crucial to determine the user’s physical status and minimize the potential impact of acute exacerbations from respiratory diseases such as pneumonia, emphysema, and pulmonary embolism.

The demand for wearable devices to measure respiratory activity continues to grow, finding applications in various settings (e.g., clinical environments and workplaces, outdoors for monitoring sports activities, etc.). The respiration rate (RR) is vital since it may represent an early marker of serious illness (e.g., pneumonia, emphysema, pulmonary embolism, etc.). Wearables such as morphic sensors may have a role in better characterising and understanding cardiorespiratory and sleep, and, within the framework of precision medicine, ultimately improve health, safety, and well-being for people with cardiorespiratory disease via non-invasive monitoring and early detection of marked cardiorespiratory changes that may be a marker of ill health.

This paper evaluates a novel electro-resistive band morphic sensor-based wearable device designed for vital signal monitoring, specifically targeting respiratory parameters. The results from this research confirmed that the morphic sensors accurately measure RR and IBI intervals with a degree of accuracy comparable to or superior to traditional polysomnography sensors.

One of the limitations of the proposed method is that artefact and body movements can impact the performance of respiratory parameter calculations. The sensor’s position is also crucial, and body movements can adversely affect their performance. For this paper, we have used our artefact detection method, “Groundtruth GUI” to identify body movement artefacts and pre-process the signal. Hence, the RR and IBI calculated from the method showed greater efficiency. In the future, we plan to integrate and automate “Groundtruth GUI” to automate the whole process. Body position did not affect the outcome of RR and IBI calculations.

In conclusion, we can infer that morphic sensors are designed with user comfort as a priority. Further work is warranted to investigate morphic sensors’ potential utility, performance, and limitations across different populations beyond those with sleep-disordered breathing.

## Figures and Tables

**Table 1:** Bill of material comparison.

Component Type	ERB Front-End Mark I [28]	ERB Front-End Mark II [27]	ERB Front-End Mark III
Instrumentation amplifier	4 (INA118)	1 (INA116)	none
Current bias generator	2 (REF200)	1 (REF200)	3 (LT3092)
Operational amplifier	4 (OPA129)	none	3 (OPA140)
Power supply	1 (DHC10512D)	1 (DHC10512D)	none
Passive resistors	12 (several different values)	1 (5 kΩ)	12 (several different values)
Capacitors	1 (2.2 µF); 2 (10 µF); 16 (100 nF)	1 (2.2 µF); 2 (10 µF); 2 (100 nF)	18: 9 (100 nF) 3 × 3 (several different values)
ERB	4 (1 m)	1 (1 m)	3 (bespoke)

**Table 2:** Anthropometric data expressed as mean ± SD.

Anthropometric Variables	Values
Age (years)	42.2 ± 12.5
Height (cm)	162.3 ± 15.7
Weight (kg)	87.3 ± 16.5
BMI (kg m^−2^)	32.9 ± 1

**Table 3:** Anthropometric data expressed as mean ± SD.

Subject Number	Morphic Sensor Respiration Rate (Breaths/Min)	Airflow (PSG) Respiration Rate (Breaths/Min)	Subject Number	Morphic Sensor Respiration Rate (Breaths/Min)	Airflow (PSG) Respiration Rate (Breaths/Min)
1	13.07 ± 0.08	13.09 ± 0.07	17	14.56 ± 0.09	14.12 ± 0.08
2	16.75 ± 0.12	16.70 ± 0.10	18	15.68 ± 0.10	16.34 ± 0.07
3	13.12 ± 0.21	13.15 ± 0.18	19	14.34 ± 0.12	13.34 ± 0.10
4	14.33 ± 0.06	14.32 ± 0.08	20	13.78 ± 0.10	14.56 ± 0.09
5	13.69 ± 0.09	13.72 ± 0.08	21	17.69 ± 0.07	16.98 ± 0.06
6	19.03 ± 0.03	19.04 ± 0.04	22	21.98 ± 0.02	21.54 ± 0.04
7	20.08 ± 0.04	20.10 ± 0.05	23	23.04 ± 0.03	23.21 ± 0.05
8	21.23 ± 0.08	19.89 ± 0.09	24	15.15 ± 0.14	16.13 ± 0.10
9	20.14 ± 0.02	21.06 ± 0.04	25	16.76 ± 0.10	17.12 ± 0.09
10	21.56 ± 0.08	20.89 ± 0.06	26	18.13 ± 0.04	17.56 ± 0.06
11	16.78 ± 0.18	15.64 ± 0.10	27	19.17 ± 0.04	19.15 ± 0.02
12	17.09 ± 0.12	16.07 ± 0.08	28	19.26 ± 0.09	19.10 ± 0.08
13	18.43 ± 0.10	19.01 ± 0.05	29	20.19 ± 0.03	21.12 ± 0.05
14	19.01 ± 0.07	18.99 ± 0.08	30	22.10 ± 0.01	22.56 ± 0.03
15	21.04 ± 0.03	21.36 ± 0.05	31	18.16 ± 0.07	19.12 ± 0.08
16	21.78 ± 0.02	20.98 ± 0.04	32	17.68 ± 0.11	18.34 ± 0.10

**Table 4:** IBI variability (mean ± SD) of pre-processed (Groundtruth GUI) data.

Subject Number	Morphic Sensor IBI Variability	Airflow (PSG) IBI Variability	Subject Number	Morphic Sensor IBI Variability	Airflow (PSG) IBI Variability
1	1.87 ± 0.01	1.78 ± 0.02	17	2.09 ± 0.01	2.12 ± 0.01
2	1.73 ± 0.02	1.67 ± 0.03	18	2.39 ± 0.01	2.23 ± 0.01
3	1.92 ± 0.01	1.93 ± 0.02	19	2.44 ± 0.01	2.32 ± 0.01
4	1.45 ± 0.03	1.38 ± 0.04	20	2.56 ± 0.01	2.45 ± 0.01
5	1.56 ± 0.03	1.67 ± 0.02	21	1.98 ± 0.02	2.06 ± 0.03
6	1.63 ± 0.01	1.60 ± 0.02	22	1.76 ± 0.02	1.74 ± 0.02
7	1.71 ± 0.01	1.63 ± 0.04	23	1.55 ± 0.04	1.56 ± 0.02
8	1.67 ± 0.02	1.58 ± 0.03	24	2.34 ± 0.01	2.32 ± 0.01
9	1.78 ± 0.01	1.73 ± 0.02	25	2.43 ± 0.01	2.41 ± 0.01
10	1.64 ± 0.03	1.63 ± 0.03	26	2.61 ± 0.01	2.63 ± 0.02
11	1.73 ± 0.01	1.74 ± 0.01	27	2.47 ± 0.02	2.43 ± 0.03
12	1.74 ± 0.01	1.81 ± 0.01	28	2.34 ± 0.02	2.31 ± 0.02
13	1.63 ± 0.02	1.56 ± 0.04	29	2.14 ± 0.03	2.12 ± 0.02
14	1.61 ± 0.04	1.73 ± 0.03	30	2.16 ± 0.02	2.24 ± 0.01
15	1.48 ± 0.04	1.54 ± 0.03	31	2.09 ± 0.03	2.12 ± 0.02
16	1.65 ± 0.01	1.62 ± 0.03	32	2.34 ± 0.02	2.33 ± 0.02

**Table 5:** Summarized scientific works based on work, sensor’s location, signal processing method, respiratory parameter computation, and accuracy.

	Sensor Type	Signal Processing Method	Respiratory Parameter Computed	Accuracy
Our proposed method	Morphic sensor	Groundtruth (artefact removal) and Peak detection	RR and IBI	95%
Huang et al. [35].	Accelerometer	Peak detection	RR	95%
Antony Raj et al. [36]	Accelerometer	Peak detection	RR	97.4%
Jarchi et al. [37]	Accelerometer	Singular Spectral Analysis (SSA) and Fast Fourier Transform (FFT)	RR	NA
Dan et al. [38]	CO_2_	Peak detection	RR	99.8%
Manoni et al. [39]	Photoplethysmography (PPG)	Power Spectral Density (PSD), Periodic Waveform Analysis (PWA)	RR	93%
Wang et al. [40]	Accelerometer and gyroscope	Variance Characterisation Series (VCS), Kalman Filter	RR	NA
Jafari Tadi et al. [41]	Seismocardiogram (SCG)	Peak detection, FFT	RR	99%

## Data Availability

Selected de-identified data can be made available upon reasonable request.

## Supplementary Materials

The following supporting information can be downloaded at: https://www.mdpi.com/article/10.3390/bios13070703/s1, Figure S1: Bland–Altman plots—RR. Difference between morphic sensor data and polysomnography airflow breaths/min plotted against the average value of the two methods. Solid horizontal lines indicate mean difference and upper/lower limits of agreement, sd: standard deviation. Figure S2: Bland–Altman plot—IBI variability. Difference between morphic sensor data and polysomnography airflow breaths/min plotted against the average value of the two methods. Solid horizontal lines indicate mean difference and upper/lower limits of agreement, sd: standard deviation.
