# Supplementary material for: Morphic Sensors for Respiratory Parameters Estimation: Validation against Overnight Polysomnography
Source: Biosensors (Basel). 2023 Jul 3;13(7):703. doi: 10.3390/bios13070703 (PMC10377422; doi:10.3390/bios13070703)
Supplement: Supplementary file 1 [file biosensors-13-00703-s001.zip › biosensors-2426789-supplementary.pdf]

Bland–Altman plots—respiratory rate (RR) and inter-breath interval (IBI) variability for morphic sensor and polysomnography.

Bland–Altman Plot— RR for morphic sensor and polysomnography: Unprocessed Data

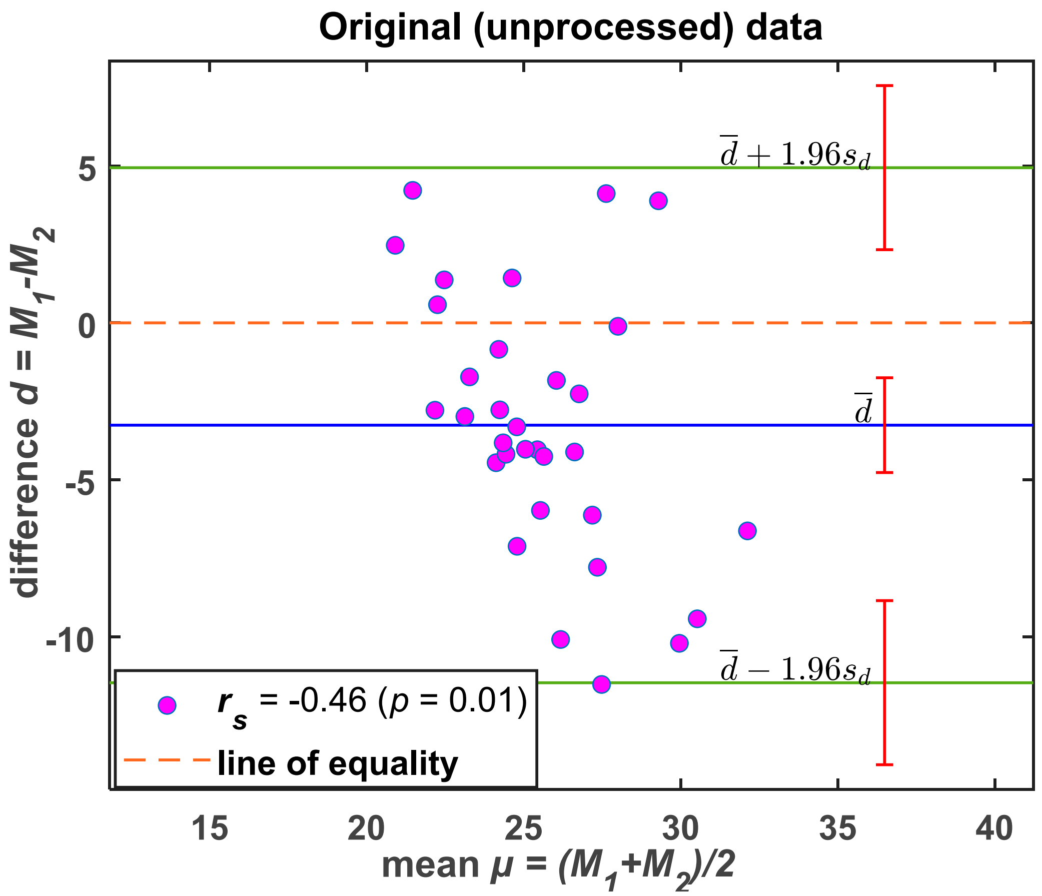

Figure S1: Bland–Altman plots—RR. Difference between morphic sensor data and polysomnography airflow breaths/min plotted against the average value of the two methods. Solid horizontal lines indicate mean difference and upper/lower limits of agreement, sd: standard deviation.

<u>Bland–Altman plot—IBI variability for</u> morphic sensor and polysomnography<u>:</u>
<u>Unprocessed Data</u>

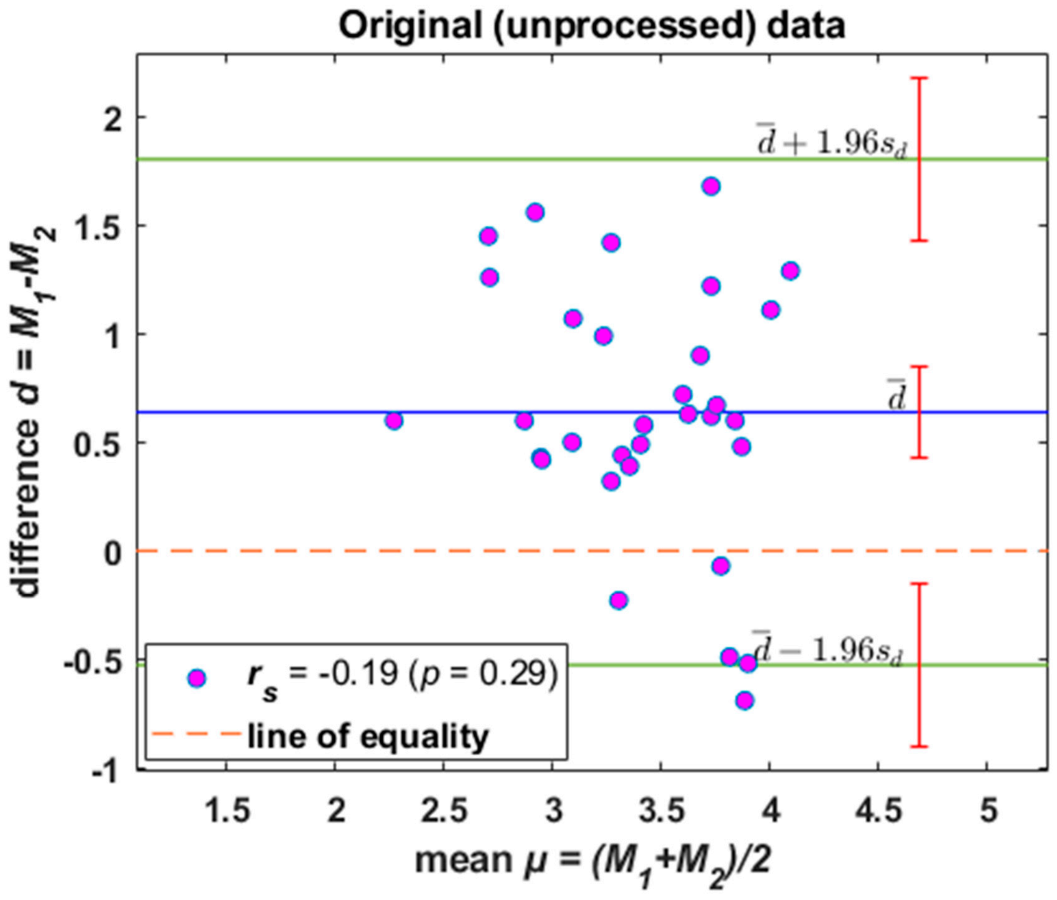

Figure S2: Bland–Altman plot—IBI variability. Difference between morphic sensor data and polysomnography airflow breaths/min plotted against the average value of the two methods. Solid horizontal lines indicate mean difference and upper/lower limits of agreement, sd: standard deviation.
